# Novel Fermented Plant-Based Functional Beverage: Biological Potential and Impact on the Human Gut Microbiota †

**DOI:** 10.3390/foods14030433

**Published:** 2025-01-28

**Authors:** Catarina Vila-Real, Célia Costa, Ana Pimenta-Martins, Samuel Mbugua, Sawadogo-Lingani Hagrétou, Kati Katina, Ndegwa H. Maina, Elisabete Pinto, Ana M. P. Gomes

**Affiliations:** 1 CBQF—Centro de Biotecnologia e Química Fina—Laboratório Associado, Escola Superior de Biotecnologia, Universidade Católica Portuguesa, Rua Diogo Botelho 1327, 4169-005 Porto, Portugal; cvreal@ucp.pt (C.V.-R.); cfcosta@ucp.pt (C.C.); apimenta@ucp.pt (A.P.-M.); ecbpinto@ucp.pt (E.P.); 2 Department of Food Science, Nutrition and Technology, University of Nairobi, P.O. Box 29053, Nairobi 00625, Kenya; s.mbugua@uonbi.ac.ke; 3 Département Technologie Alimentaire (DTA), Institut de Recherche en Sciences Appliquées et Technologies (IRSAT), Centre National de la Recherche Scientifique et Technologique (CNRST), Ouagadougou 03 BP 7047, Burkina Faso; hagretou@yahoo.fr; 4 Department of Food and Nutrition, University of Helsinki, Agnes Sjöbergin katu 2, 00014 Helsinki, Finland; kati.katina@helsinki.fi (K.K.); henry.maina@helsinki.fi (N.H.M.); 5 EPIUnit—Instituto de Saúde Pública, Universidade do Porto, Rua das Taipas 135, 4050-600 Porto, Portugal

**Keywords:** fermented cereal, gut microbiota, symbiotics, synbiotic, lactic acid bacteria, finger millet

## Abstract

Controlled fermentation carried out by selected starters might enhance the safety, nutritional, and biological profiles of non-dairy fermented products. This research aims to study the biological potential and impact on the human gut microbiota of a novel fermented finger millet-based product. Finger millet (*Eleusine coracana*), suspended in an aqueous sucrose-based solution, was fermented by *Weissella confusa* 2LABPT05 and *Lactiplantibacillus plantarum* 299v (1%, 1:1 ratio (*v*/*v*)), at 30 °C/200 rpm in an orbital incubator until pH ≈ 4.5–5.0. Microbial growth, phenolic compounds, antioxidant, and antidiabetic activities were evaluated. In vitro digestion followed by in vitro faecal fermentation were used to study the impact of the fermented plant-based functional beverage (PBFB) on the human gut microbiota. Antidiabetic activity (21% *vs*. 14%) and total phenolics (244 *vs*. 181 mg of gallic acid equivalents/kg PBFB) increased with fermentation. The digested fermented PBFB contributed to the increase, over the first 6 h, of the *Bifidobacterium*’s 16S rRNA gene copy numbers, concomitant with significant release of the acetic, propionic, and butyric short chain fatty acids, and also lactic acid. The novel PBFB has been shown to have antidiabetic potential and bifidogenic effects, and consequently its consumption might positively impact blood glucose levels and the human gut microbiota.

## 1. Introduction

Non-dairy fermented products are attracting increasing market interest due to consumers’ openness to new concepts, growing health concerns—such as the ‘free-from’ trend and the search for alternative products due to gluten and lactose intolerances—and rising awareness of environmental and animal welfare issues [1,2,3]. In response, the development of functional probiotics cereal-based products is increasing [4]. Lactic acid bacteria (LAB) have long been utilised as starter cultures, effectively enhancing product quality, safety, shelf-life, and nutritional and sensory properties [5,6]. In addition, well-controlled LAB-fermented food products often exhibit positive biological effects, in particular when probiotic organisms such as *Bifidobacterium* and/or specific *Lactobacillus* species are incorporated [7]. The impact of fermentation on antioxidant activity and total phenolic content has been investigated, yielding controversial results [8,9,10,11,12,13]. These variations are influenced by factors such as pre-fermentation processing steps and the specific characteristics of the matrix. The potential of fermented foods having antihyperglycemic activity has also been studied, and interesting results were observed for mung [14,15], soy [16], beans, and leaf tea [17], and also for finger millet [18,19].

Over recent years, the literature has been conducive to the importance of including fermented foods in a daily diet [1,20,21]. Apart from the above-mentioned positive bioactivities of fermented plant-based products, it seems that those foods have a positive impact on the human gut modulation. The review of Singh and colleagues (2022) was particularly interesting in this context, focusing on the health benefits, mainly the antidiabetic effects, of finger millets and the role of its bioactive compounds on the gut microbiota, specifically against diabetes [22]. Probiotics impact gastro-intestinal well-being by promoting the growth of beneficial bacteria and neutralising the harmful species, which helps the regeneration of the gut microbiota [23]. The predominant phyla in the adult gut microbiota include Firmicutes, Bacteroidetes, Verrucomicrobia, Proteobacteria, and Actinobacteria [24,25]. The activity of gut microbial community results in the production of short-chain fatty acids (SCFA), such as butyrate, propionate, and acetate, which play a key role in lipid metabolism and strengthen the mucosal barrier and defence [24,26]. Other reported health-promoting functions are the regulation of glucose homeostasis, the modulation of immune and inflammatory responses, the biosynthesis of de novo vitamins and amino acids, and appetite regulation [24,27,28,29]. Conversely, an imbalance of specific bacterial groups has been linked to conditions such as colorectal cancer, inflammatory bowel disease, irritable bowel syndrome, and diabetes [25,30,31,32]. Although microbial modulation offers positive health effects, it is well-established that changes in the microbial community are transient and that the composition of the human microbiome adapts at an individual rate [33,34]. Significant advances have been made in recent years [24,25], yet further research is essential to deepen our understanding of the complexity, composition, and functions of the gut microbiota, often referred to as the ‘hidden human organ’.

Based on the above-mentioned rationale, the authors hypothesised that a novel fermented plant-based beverage developed using a gluten-free whole-grain cereal might possess not only particular bioactivities, but also potential for the modulation of the human gut microbiota. Towards the assessment of this hypothesis, a finger millet-based product was fermented by a bacterial consortium including a probiotic strain and an exopolysaccharides-producing strain, developing a beverage which was studied for its potential antioxidant and antidiabetic activities and its impact on the human gut microbiota, using an in vitro digestion model followed by a fermentability assay. Finger millet was specifically chosen for this research due to its rich nutrient profile, namely including being gluten-free, high in fibre, rich in bioactive compounds, and a relevant source of micronutrients [35]. Additionally, its advantageous environmental characteristics contributed to its selection, as it is drought-resistant, well-adapted to semi-arid and arid regions, and requires minimal harvesting resources [35]. As far as the authors are aware, the use of finger millet as a substrate for fermentation by a carefully selected bacterial consortium, including probiotics and techno-functional cultures, remains underexplored. This study bridges this gap by providing novel insights into the potential of LAB-fermented finger millet as a functional food with gut health benefits.

## 2. Materials and Methods

### 2.1. Sample Preparation

Cleaned finger millet (*Eleusine coracana* (L.) Gaertn) grains, sourced from a local producer in Nairobi, Kenya, underwent a multistep milling process. Initially, the grains were milled using industrial milling equipment (CD1 mill, Chopin Technologies, KPM Analytics, Villeneuve-la-Garenne, France), followed by further milling with a lab-scale Thermomix (Type TM5-2, Vorwerk Elektrowerke GmbH & Co. KG, Wuppertal, Germany). Finally, a mortar and pestle were used to reduce any remaining particles larger than 500 μm, achieving a final granulometry of <500 μm. Dry-milled finger millet flour was sterilised (121 °C, 15 min) and subsequently suspended in a sterile aqueous sucrose-based solution (10% (*w*/*v*)) (1:9 (*w*/*w*)). These suspensions, referred to hereafter as slurries, were prepared in triplicate. Non-inoculated slurries were prepared and monitored under the same fermentation conditions to serve as controls.

### 2.2. Microorganisms, Growth Conditions, and Inoculation

A co-culture including *Lactiplantibacillus plantarum* 299v (obtained from PROBI AB, Lund, Sweden) and *Weissella confusa* 2LABPT05 (isolated from a traditionally fermented pearl millet pancake—*Massa*—from Burkina Faso) was used. The microorganisms, stored at −80 °C, were individually reactivated by inoculating 2% (*v*/*v*) into Man–Rogosa–Sharpe (MRS) broth (Biokar Diagnostics, Beauvais, France), and incubating overnight at 37 °C. The inoculum was subsequently propagated at 1% in MRS broth, for at least two successive transfers, with 24 h intervals, until the day of inoculation. On the day of experiment, both strains (*L*. *plantarum* 299v at 10^9^ CFU/mL, and *W*. *confusa* 2LABPT05 at 10^7^ CFU/mL)) were mixed in a 1:1 (*v*:*v*) ratio and inoculated at 1% (*v*/*v*) in each slurry.

### 2.3. Fermentation Process and Enumeration of Microorganisms

The fermentation process was carried out in an orbital incubator (Bench Top Shaking Incubator, Wiggen Hauser, Berlin, Germany) at 30 °C and 200 rpm, for 8 h. Aliquots (2 mL) were collected at different time points of the fermentation process (before inoculation and at 0, 4, 6, 8 h of fermentation), for chemical analysis, including pH, organic acids and sugars concentrations, as well as for assessing the microbial growth of lactobacilli, *Weissella*, and potential contaminant bacteria. The final fermented product is hereafter referred to as the plant-based functional beverage (PBFB).

A differential medium (De Man, Rogosa, and Sharpe (MRS; Biokar Diagnostics, Beauvais, France) agar supplemented with bromophenol blue (BPB)) was used for individual counts of each bacterium, whose preparation was based on the study by Lee and Lee (2008) [36]. Also, plate count agar (PCA; Biokar Diagnostics, Beauvais, France) was used, for the total bacterial count. In order to confirm the microbiological quality of the cereal slurries (absence of contaminants), potato dextrose agar (PDA) (Biokar Diagnostics, Beauvais, France) was prepared for the enumeration of yeasts and moulds, and violet red bile glucose agar (VRBGA), was prepared for the enumeration of enterobacteriaceae (BioMérieux, Craponne, France). These two media were only used before the inoculation of the strains. All media plates were prepared in duplicate. Microorganisms were enumerated according to Miles et al. (1938) [35], following appropriate incubation at 37 °C for longer than 72 h in the case of PCA plates and for 48 h for MRS and VRBGA, and at 30 °C for 48 h for PDA. The results were expressed in colony forming units (CFU)/mL.

The pH of samples was measured at each time point at room temperature, using a pH metre equipped with a pH electrode (Crison Micro pH 2002, Barcelona, Spain). Sugars and organic acids concentrations were simultaneously determined via high-performance liquid chromatography (HPLC) with the refractive index and ultra-violet detection, respectively. The HPLC analysis followed the method described by Sousa et al. (2015) [37], with the following modifications: approximately 2 g of each sample were diluted in 5 mL of 13 mM sulphuric acid (95–97%, Merck KGaA, Darmstadt, Germany), homogenised with an Ultra-Turrax homogeniser (T18 Basic; IKA Works, Inc., Wilmington, NC, USA) at 18,000 rpm for 3 min, filtered through no. 1 filter paper (Prat Dumas France, 2–4 μm), and subsequently filtered through 0.45 μm pore-size filters (Chromafil^®^ PET—45/25, Macherey-Nagel, Germany) immediately prior to injection. Each sample was injected once, and the data were collected and analysed using Clarity system software (version 5.0.5.98). Quantification was performed using the calibration curves of the appropriate chromatographic standards.

### 2.4. Biological Activity of the Fermented Product

Within biological activity, the determination of total phenolics content and the assessment of antioxidant and antidiabetic activities potential were envisaged.

#### 2.4.1. Total Phenolic Content and Antioxidant Activity

Total phenolic content was determined using the Folin–Ciocalteau method, as expressed as gallic acid equivalents (mg/L) [38]. Prior to this analysis, the exopolysaccharides (EPS) produced during the fermentation process were removed once they would interfere with the spectrophotometry analysis. EPS were precipitated with cold ethanol (1:1, *v*:*v*) overnight, followed by centrifugation at 4,000 rpm at 4 °C for 20 min. The supernatant was recovered and used in further analyses. Unfermented (aliquots taken at time 0 h) and fermented (aliquots taken at time 8 h) samples were studied. Briefly, 50 µL of Folin–Ciocalteau reagent (Merck, KGaA, Darmstadt, Germany), 1 mL of 75 g/L sodium carbonate (Sigma-Aldrich) and 1.4 mL of deionized water were sequentially added to 50 µL of the sample (supernatant). Then, the samples were agitated using a vortex and allowed to react for 1 h, away from the light. Absorbances were read at 750 nm using a Shimadzu UV mini-1240 spectrophotometer, with measurements performed in triplicate.

The antioxidant activity was evaluated using two methods: ABTS (2,2-Azinobis, 3-ethylbenzthiazoline-6-sulphonic acid) and DPPH (2,2-diphenyl-1-picrylhydrazyl). While both methods rely on electron transfer and involve redox reactions, the DPPH assay, being insoluble in water, is more efficient for measuring less polar compounds. In contrast, the ABTS assay, which is soluble in both aqueous and organic (alcoholic) solvents, is better suited for water-soluble compounds.

The ABTS assay followed the methodology proposed by Re et al. (1999) [39], with modifications by Gião et al. (2007) [40]. The ABTS^•+^ solution was prepared by mixing 7 mmol/L ABTS diammonium salt (Sigma-Aldrich) solution with 2.45 mmol/L potassium persulfate (Merk, KGaA, Darmstadt, Germany) solution, at a 1:1 (*v*:*v*) ratio. This reaction mixture was stirred for 16 h at room temperature in the dark. The resulting solution was stored at 4 °C until further use. On the day of analysis, the aforementioned ABTS^•+^ solution was diluted in deionised water to achieve an absorbance of 0.700 ± 0.020, at 734 nm (UV–Vis spectrophotometer, UVmini 1240, Shimadzu, Tokyo, Japan). For the assay, 10 μL of each sample were added to 1 mL of diluted ABTS^•+^ solution, and the absorbance was measured at 734 nm after 6 min. Each sample was analysed in triplicate. A calibration curve was prepared using ascorbic acid as the standard, and the results were expressed as the equivalent concentration of ascorbic acid (g/L). Also, the antioxidant capacity was calculated as the percentage of inhibition (PI), according to the following equation:

$$\mathrm{PI}=\frac{{\mathrm{Abs}}_{{\mathrm{ABTS}}^{•+}}-{\;\mathrm{Abs}}_{\mathrm{sample}}}{{\mathrm{Abs}}_{{\mathrm{ABTS}}^{•+}}}\;\times 100,\;$$

where Abs_ABTS_^•+^ corresponds to the initial absorbance of diluted ABTS^•+^ and Abs_sample_ corresponds to the absorbance of the sample after 6 min of reaction.

The DPPH assay was performed following the method of Bondet et al. (1997) [41], with minor modifications. A concentrated DPPH^•^ solution was prepared by dissolving DPPH (Sigma-Aldrich) in methanol (Honeywell, Riedel-de Haen™) at a concentration of 600 µM. This solution was stored at −20 °C and protected from light until further use. On the day of analysis, the aforementioned DPPH^•^ concentrated solution was diluted tenfold to achieve an absorbance of 0.600 ± 0.100 at 515 nm (UV–Vis spectrophotometer, UVmini 1240, Shimadzu, Tokyo, Japan). For the assay, 250 μL of the sample was mixed with 1.75 mL of the diluted methanolic DPPH^•^ solution and allowed to react for 30 min in the dark. The absorbance was then measured at 515 nm. Each sample was read in triplicate. A calibration curve was prepared using Trolox as the standard, and the results were expressed as the equivalent concentration of Trolox (g/L). Additionally, the antioxidant capacity was calculated as the percentage of inhibition, according to the following equation:

$$\mathrm{PI}=\frac{{\mathrm{Abs}}_{{\mathrm{DPPH}}^{•}}-{\mathrm{Abs}}_{\mathrm{sample}}}{{\mathrm{Abs}}_{{\mathrm{DPPH}}^{•}}}\;\times 100$$

where Abs_DPPH_^•^ corresponds to the initial absorbance of DPPH^•^ solution and Abs_sample_ corresponds to the absorbance of the sample after 30 min of reaction.

#### 2.4.2. Antidiabetic Activity—α-Glucosidase Inhibitory Activity

The samples used for this analysis were the same as those used for organic acids and sugars determination. Unfermented and fermented samples were studied. The α-glucosidase inhibitory activity was determined in 96-well plates according to the method described by Kwon et al. (2008) [42]. The supernatant (50 μL), at the concentration of approximately 30 mg cereal flour/mL, was mixed with 100 μL of 0.1 M phosphate buffer (pH 6.9) containing α-glucosidase solution (1.0 U/mL), and pre-incubated at 25 °C for 10 min. Then, 50 μL of 5 mM p-nitrophenyl-α-D-glucopyranoside solution in 0.1 M phosphate buffer (pH 6.9) was added to each well at 5-s intervals. The reaction mixtures were incubated at 25 °C for 5 min, and the absorbance readings were recorded at 405 nm by a multiscan microplate fluorimeter (FLUOstar Optima, BMG Labtech, Offeuburg, Germany) and compared to a control which had 50 μL of H_2_SO_4_ 13 mM in place of the sample. Acarbose (Sigma) was used as a positive control, at the concentration of 10 mg/mL. The α-glucosidase inhibitory activity was expressed as the percentage of inhibition, and was calculated as follows: $\mathrm{PI}=\frac{{\Delta Abs}_{control}-{\Delta Abs}_{sample}}{{\Delta Abs}_{control}}\;\times 100,$ where ΔAbs_control_ is the variation in absorbance of the control and ΔAbs_sample_ is the variation in absorbance of the samples, between the beginning level and after 5 min of reaction.

### 2.5. In Vitro Simulation of Gastrointestinal Digestion of the Fermented Product

#### 2.5.1. Simulated Digestion Fluids

In order to simulate the digestion of the fermented product, the standardised static in vitro digestion method, advanced by Minekus et al. (2014) [43], specifically the protocol developed from it [44], was followed. All the simulated digestion fluids (simulated salivary fluid (SSF), simulated gastric fluid (SGF), and simulated intestinal fluid (SIF)), CaCl_2_ (Honneywell Fluka), and water were previously prepared. Enzymatic solutions were prepared on the day of the experiment:

- Salivary α-amylase (α-amylase from human saliva Type XIII-A, lyophilized powder, 300–1500 U/mg protein, Sigma) solution, at 75 U/mL in the final mixture, was made up in SSF electrolyte stock solution.
- Porcine pepsin (pepsin from porcine gastric mucosa, powder, ≥250 U/mg solid, Sigma), at 2000 U/mL in the final mixture, was made up in SGF electrolyte stock solution.
- Pancreatin (pancreatin from porcine pancreas, powder solution), at 100 U/mL, and bile salts (Oxoid Limited, Thermo Fisher (Heysham) Limited, Lancaster, United Kingdom), at 12 g/L, were made up in SIF electrolyte stock solution.

#### 2.5.2. Oral, Gastric, and Intestinal Phases

After the 8 h fermentation process, the obtained liquid fermented product (the so-called fermented PBFB) was freeze-dried without cryoprotectants. To simulate the oral phase, water was added to this freeze-dried fermented product (1:2, *w*:*w*), followed by the addition of the SSF electrolyte stock solution (800 μL/mL sample), CaCl_2_ (5 μL/mL sample), salivary α-amylase solution (195 μL/mL sample) and water (final ratio of food to SSF of 50:50). The mixture was thoroughly mixed and incubated for 2 min at 37 °C, at 200 rpm in an orbital incubator. To simulate the gastric phase, SGF electrolyte stock solution (800 μL/mL oral bolus), CaCl_2_ (0.5 μL/mL oral bolus), pepsin solution (50 μL/mL oral bolus), HCl (to reach pH 3.0), and water were sequentially added to the previous oral bolus (final ratio of oral bolus to SGF of 50:50). The mixture was thoroughly mixed and incubated for 2 h at 37 °C, at 200 rpm in an orbital incubator. After gastric digestion simulation, the intestinal phase was mimicked. Next, SIF electrolyte stock solution (425 μL/mL gastric chyme), CaCl_2_ (2 μL/mL gastric chyme), pancreatin solution (250 μL/mL gastric chyme), bile solution (125 μL/mL gastric chyme), NaOH (to reach pH 7.0), and water were sequentially added to the gastric chyme (final ratio of oral bolus to SIF of 50:50). The mixture was thoroughly mixed and incubated for 3 h at 37 °C, at 200 rpm in an orbital shaker.

Afterwards, the absorption in the small intestine was simulated by including a step of dialysis. The digested fermented PBFB (DF-PBFB) was transferred to 1 kDa molecular weight cut-off regenerated cellulose dialysis tubing (Spectra/Por 6 dialysis tubing, 1 kDa, 45 mm flat-width, 10 m/roll, Spectrum Europe, Netherlands) and dialysed against NaCl 0.01 M to remove low molecular mass digestion products, at room temperature, at 500 rpm in a magnetic plate stirrer. After 15 h, the fluid was changed and dialysis continued for two more hours, after which the content was transferred to freeze-drying recipients and DF-PBFB was freeze-dried, aiming to produce a powder to be used for the next phase, the faecal culture fermentations.

Cell viability was evaluated after the digestion process simulation. Suitable dilutions of the DF-PBFB suspension were plated on MRS agar supplemented with BPB and PCA, using the Miles technique, as previously described. Also, free *L*. *plantarum* 299v and *W*. *confusa* 2LABPT05 suspensions were monitored in order to study the impact of the cereal matrix carrier on strains’ viability.

### 2.6. Faecal Fermentations

#### 2.6.1. Recruitment of Participants and Collection of Samples

Healthy adult volunteer donors (*n* = 5) were purposively recruited in the Escola Superior de Biotecnologia, Universidade Católica Portuguesa. The eligibility criteria included being a healthy adult between 18 and 40 years old, to have an omnivorous diet, not having any food intolerances/allergies, and having not consumed prebiotic or probiotic supplements (including ‘yoghurts with bifidus’) or antibiotics in the six months prior to the experiment. All subjects were informed about the study’s objectives, and those who agreed to participate signed an informed consent form that provided detailed information on the conduct of the study, in accordance with the Declaration of Helsinki. Additionally, each participant received comprehensive instructions for faecal sample collection, along with the necessary materials, following an internally established protocol, previously validated by the Health Ethics Committee of the Universidade Católica Portuguesa (project No. 167). Each participant collected their sample in a clean container, which was immediately sealed after placing an anaerobic sachet inside, along with the sample.

#### 2.6.2. Faecal Fermentation Conditions and Procedure

A basal nutrient medium, with a pH 7.0 (peptone water 2 g/L, yeast extract 2 g/L, NaCl 0.1 g/L, K_2_HPO_4_ 0.04 g/L, KH_2_PO_4_ 0.04 g/L, MgSO_4_·7H_2_O 0.01 g/L, CaCl_2_·6H_2_O 0.01 g/L, NaHCO_3_ 2 g/L, Tween 80 2 mL/L, hemin 0.05 g/ L, vitamin K 10 μL/L, L-cysteine HCl 0.5 g/L, bile salts 0.5 g/L, and resazurin 4 mg/L) was previously prepared, according to the procedure outlined in [45]. Three independent fermentations, with independent tubes for each sampling time, were run in parallel:

(1) The digested fermented PBFB.
(2) A positive control (C+), FOS from chicory root, purity: >95%, degree of polymerization between 2 and 8 (Megazyme, Bray, Ireland).
(3) A negative control (C−) which had no source of carbon added (instead of the sample, the basal medium was added).

The DF-PBFB and the C+ were suspended at 2% (*w*/*v*) in the basal nutrient media and then dispensed to the tubes (9.8 mL), in an anaerobic workstation (Don Whitley Scientific, West Yorkshire, UK), which was maintained at 37 °C under an anaerobic atmosphere (10% CO_2_, 5% H_2_, and 85% N_2_). For the C−, the medium was directly dispensed to the tubes (9.8 mL). Faecal samples were used within 30 min of collection. Five independent faecal inocula were prepared in a phosphate-buffered saline (PBS) solution (10%, *w*/*v*), in the above-cited anaerobic atmospheric conditions. Faecal inoculum was added at 2% to each tube containing the basal medium.

Samples were collected at different time points (0, 6, 12, and 24 h after inoculation) for pH control during fermentation. Moreover, these sampling aliquots were used for the determination of organic acids and sugars concentration and the extraction of genomic DNA (gDNA).

#### 2.6.3. Acidification and Organic Acids Production

The pH of independent tubes (C+, C−, and DF-PBFB) was measured, at every time point, at room temperature using a pH metre equipped with a pH electrode (Crison Micro pH 2002, Barcelona, Spain). Samples were then stored at −20 °C until further analyses. The previously frozen samples were defrosted, and aliquots of 2 mL were centrifuged (Boeco Centrifuge U-320R, Hamburg, Germany) at 4000× *g*, for 10 min. The supernatant was used for the evaluation of organic acids production, according to the operational conditions previously described. The pellet was further used for gDNA extraction.

### 2.7. Bacterial Enumeration of the Gut Microbiota

#### 2.7.1. DNA Extraction and Quantification

DNA was extracted using the NZY Tissue gDNA Isolation kit (NZYTech, Lisbon, Portugal), and some adaptations were made to the standard manufacturers’ protocol [46]. To the previously mentioned pellet, a 2 mL aliquot of the sample was added and another centrifugation was carried out, at 4000× *g*, for 10 min. The supernatant was discarded, and then the pellet was washed with 1 mL of TE buffer (10 mM Tris/HCl; 0.1 mM EDTA, pH 8.0, previously prepared), vortexed, and centrifuged at 4000× *g* for 10 min. This process was repeated until the obtention of a colourless supernatant. To promote the pre-lysis of the sample, 300 μL of buffer NT1 was used to re-suspend the sample, dissolving the pellet by an up-and-down movement with the pipette. From this, 200 μL were transferred to a new Eppendorf, which was vortexed and incubated at 95 °C for 10 min. After this incubation time, 25 μL of proteinase K were added and the samples were vortexed and incubated at 56 °C, for 1 h 45 min, vortexing after half of this time. In order to lyse the sample, samples were vortexed and 200 μL of buffer NL were added, followed by vortex agitation for 10 s and centrifugation at 11,500× *g*, for 10 min. The obtained supernatant was transferred to a new Eppendorf and the pellet was discarded. The following steps, namely the addition of ethanol, the DNA binding, the washing and drying of silica membrane, and finally the elution of the DNA, were carried out exactly according to the abovementioned protocol. Once extracted, the concentration and purity (through the analysis of Abs_260nm_/Abs_230nm_ and Abs_260nm_/Abs_280nm_ ratios) of DNA were determined using a Thermo Scientific^TM^ μDrop^TM^ Plate coupled with a Thermo Scientific^TM^ Multiskan^TM^ FC Microplate Photometer (Thermo Fisher Scientific, Waltham, MA, USA). The obtained concentration of gDNA was diluted and equilibrated to 20 ng/μL. The stock and diluted genomic DNA samples were stored at −20 °C until further analysis.

#### 2.7.2. Bacterial Enumeration Using Real-Time Quantitative-PCR

A CFX96 Touch^TM^ Real-Time PCR Detection System (Bio-Rad Laboratories, Inc., Hercules, CA, USA) was used to perform qPCR. The cycling programme of qPCR was made up of the following steps:

(1) Initial denaturation/enzyme activation, at 95 °C, for 5 min; (2) denaturation, at 95 °C, for 10 s; (3) annealing, for 1 min, at primer-specific temperature; (4) extension, at 72 °C, for 30 s; (5) melt curve generation, at 60–97 °C, increasing at an increment of 0.5 °C, for 5 s.

Steps 2, 3, and 4 comprised the amplification reaction, and they were repeated 45 times (45 cycles). The PCR reaction volume was 10 μL of the following mixture: 5 μL of 2x iQ^TM^ SYBR^®^ Green Supermix (Bio-Rad Laboratories, Inc., Hercules, CA, USA), 2 μL of ultrapure water, 1 μL of sample-diluted DNA (20 ng/μL), and 1 μL of forward and reverse primers (100 nM) targeting the 16S rRNA gene. All assays were performed in quadruplicate and, during every run, a calibration curve was constructed.

The annealing temperature of each primer (STABvida, Lisbon, Portugal) was optimised. The optimal annealing temperature was determined by testing a range of annealing temperatures above and below the calculated T_m_ of the primers of each bacterial strain. The optimal annealing temperature was then established for the PCR runs. Table 1 presents the primers sequence, and the respective optimal annealing temperature, genome size and number of copies of the 16S rRNA gene for each used bacterial strain.

### 2.8. Statistical Analysis

Paired-sample T-tests were used to study differences between each parameter before and after fermentation. A Shapiro–Wilk test was used for checking the normality of the data. In cases in which the normality of the data was not verified, the Wilcoxon non-parametric test was the alternative test used.

For the GI simulation, an independent-T test was used to detect differences in the number of viable cells between samples (free cells or those inoculated in the cereal slurries).

For the fermentation assay, a one-way ANOVA coupled with a Tukey’s post hoc test was carried out to detect significant differences in the log number of the16S rRNA gene copies obtained between samples (C−, C+, and DF-YLB) at distinct times (0 h, 6 h, 12 h, or 24 h).

The normality and homoscedasticity of data were determined by Shapiro–Wilk and Levene’s tests, respectively. In cases in which the normality and/or homoscedasticity of data were not verified, Kruskal–Wallis and Mann–Whitney non-parametric tests were the alternatives to the ANOVA and Tukey’s test/independent T-test that were used, respectively. The significance level was set at 5% (*p*-values ≤ 0.05) for all tests performed. Statistical analyses were conducted using IBM^®^ SPSS^®^ Statistics, version 26 (SPSS Inc., Chicago, IL, USA).

## 3. Results and Discussion

### 3.1. Fermentation of the Wholegrain Finger Millet Slurry

The main results relative to the fermentation of the wholegrain finger millet slurries are not presented and discussed here because they are not the focus of the present paper. Nevertheless, these results are described in detail in the study of Vila-Real et al. (2022) [47]. Briefly, an eight-hour fermentation enabled *L*. *plantarum* 299v to increase by one log cycle and reach final viable cell numbers around 10^8^ CFU/mL (above the minimum requirement of 10^7^ CFU/g [48]). On the other hand, *W*. *confusa* 2LABPT05 achieved a much more impactful increase of about four log cycles, proving that it is not negatively affected by the probiotic strain’s presence; on the contrary, its growth and metabolic activities are enhanced, and it can still perform its crucial technological role. The pH’s drop reached the final value of 4.8. The main produced organic acids were lactic and acetic acids, while mainly sucrose and glucose were utilised by the microorganisms, resulting in the release of fructose.

### 3.2. Biological Activity of the Fermented Product

#### 3.2.1. Total Phenolic Content (TPC) and Antioxidant Activity

Fermentation was shown to cause a 35% increase in the content of total phenolics (Table 2). In contrast, the DPPH scavenging activity decreased within the 8 h fermentation, from 26.7 ± 0.6% inhibition to 23 ± 1% (correspondent to the initial 0.18 ± 0.01 mg TE/ g of lyophilized extract, and final 0.15 ± 0.01 mg TE/ g of lyophilized extract concentrations, respectively). The ABTS radical scavenging activity of the F-PBFB was also slightly lower, but not statistically different, varying from 27.2 ± 0.9% to 25.6 ± 0.6%. During fermentation, the acidic environment and the increased enzymatic activity might promote the hydrolysis of the polymerised phenolic compounds, liberating simpler phenolic compounds [49,50,51]. Those enzymes may come from the cereal matrix and the existent microflora [49,50]. Rizzielo et al. (2016) also observed an increase in total phenolic content in quinoa sourdough fermented by *Lactiplantibacillus plantarum* T6B10 and *Lactobacillus rossiae* T0A16 [51]. This increase was accompanied with an augment in the DPPH activity as well, contrary to the present results. Another microbial-related mechanism that may be involved in these alterations during fermentation is non-enzymatic. It has been suggested that the *Weissella confusa*’s EPS have antioxidant activity [52,53]. Specifically, in Adebayo et al.’s study (2018) [53], in which a wild type of *Weissella confusa* was studied, the authors concluded that the antioxidant activity increased in a dose-dependent manner. In the present study, EPS were precipitated prior to the assessment of the phenolics content and antioxidant activities, and the samples in question here were the EPS-free supernatants, in order to observe the compounds released to the medium. On the one hand, this might justify the increased total phenolics content, and on the other hand, it might justify the decrease in antioxidant activity, which in this case seems to be associated with the presence of EPS.

#### 3.2.2. Antidiabetic Activity—α-Glucosidase Inhibitory Activity

The α-glucosidase inhibitory activity was improved by 7% after fermentation. This augmented inhibitory activity might be related to the increase in TPC and the release of α-glucosidase inhibitory phytochemicals; in their original form, these may be found bound to proteins, and consequently with hampered activity [54]. Furthermore, the three-fold increase in the GABA amino acid, after an 8 h fermentation period (published elsewhere [47]), may also explain these results. Han and Lee (2017) [55] found a relation between the yeast strains’ content of GABA and their demonstrated α-glucosidase inhibitory activity. The cell-free extract from the two highest GABA-producing yeasts had the highest anti-hyperglycemic α-glucosidase inhibitory activity, of 72.3%, and 69.9%. These authors further explored the anti-hyperglycemic action of the same two cell-free extract-containing GABA amino acid in normal and diabetic rats, and observed a decrease in blood glucose levels, similarly to the positive control (acarbose); nevertheless, high doses were required [55]. Additionally, Hariyanto et al. (2022) linked high levels of GABA production during the fermentation of soybean residue by the co-culture of *L. plantarum* and *Rhizopus oligosporus* with ameliorated blood glucose homeostasis, observed by *in vivo* assays [56]. Chen et al. (2013) [57] observed that some LAB strains (namely the cell-free extracts and supernatants of *L*. *plantarum*) have demonstrated potential for α-glucosidase inhibitory activity, but of a lower order of magnitude. Some other authors [58] suggest that this activity might come from the production of EPS by LAB, a mechanism which may also be in question in the present study, given the production of EPS, mainly by the *Weissella* strain.

### 3.3. In Vitro Simulation of Gastrointestinal Digestion of the Fermented Product

#### Simulation of Digestion in the Gastrointestinal Tract

**Figure 1:**
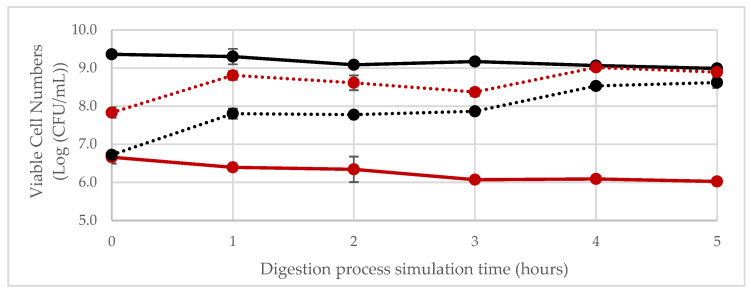
Evolution of bacterial growth of *W*. *confusa* 2LABPT05 (red lines) and *L*. *plantarum* 299v (black lines). Full lines are relative to the individual growth of the strains in free form, whereas dashed lines are relative to the growth of the strains inoculated in combination in the finger millet slurry during the simulation of digestion in the gastrointestinal tract process, at 37 °C, 200 rpm, over 5 h in an orbital incubator. Error bars represent the standard deviation of independent replicates.

The microbiological results concerning the simulation of GI digestion are presented in Figure 1. It is possible to observe that, while in the free form—namely, not embedded in any matrix—both *L*. *plantarum* 299v and *W*. *confusa* 2LABPT05 decreased by approximately 0.5 log cycles during their passage through the GI-simulated conditions of both the gastric and intestinal phases. It is well recognised that *L*. *plantarum* 299v is able to survive the passage through the gastrointestinal tract [59,60]. Also, some *Weissella* strains (*W*. *confusa* or *W*. *cibaria*) have shown higher survival rates in the presence of bile salts (0.3%) and in an acidic environment (pH = 3), showing a promising capability to successfully pass through the GI tract [61]. Nonetheless, when inoculated in the fermented PBFB*,* the results were slightly different. It seems that the cereal matrix offered a protective environment to both strains, enhancing viable cell numbers, particularly in the first hour of the gastric phase (t = 1 h) (*p* ≤ 0.05). This shows the important role of the cereal in the bacterial delivery [62]. As shown by Patel and colleagues, the physicochemical characteristics and pH of the food carrier have a significant impact on the strains’ viability [63].

### 3.4. Fermentability Assay Using Human Faecal Samples

A total of five healthy subjects were recruited as faeces sample donors, of whom two were female and three were males, with ages between 27 and 33 years old.

After the simulation of small intestinal absorption, the DF-PBFB was freeze-dried and used for faecal culture fermentation through dialysis and established for 24 h in which the DF-PBFB was placed in contact with the human faecal samples to study its effect upon the human microbiota. Aliquots from different locations on the stools were targeted, since the taxa concentration might vary from the peripheries to the centre of the stool specimen [64].

**Figure 2:**
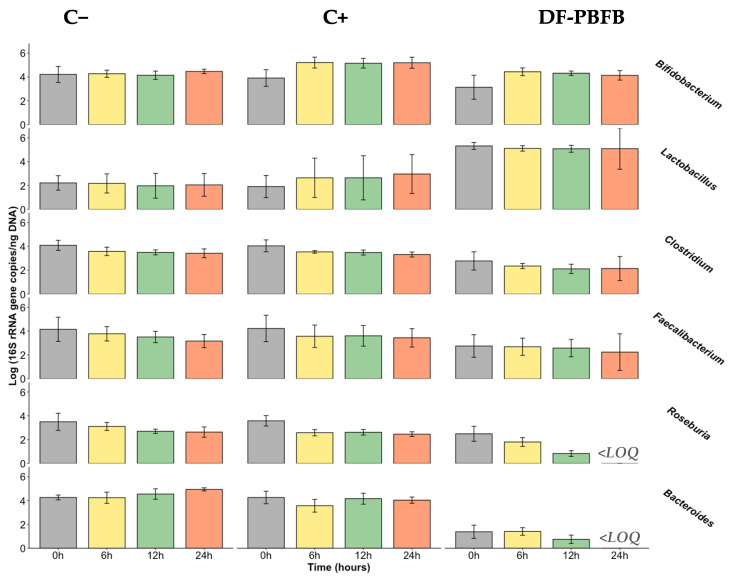
Quantification of the bacterial groups (expressed as log (16S rRNA gene copies/ng of DNA); mean ± standard deviation) detected by qPCR in faecal samples (negative control (C−), positive control (C+; FOS), digested fermented plant-based functional beverage (DF-PBFB)) of five donors. LOQ: limit of quantification.

After DNA extraction and equilibration to the final concentration of 20 ng/μL, a real-time qPCR was used to determine the number of copies of the 16S rRNA gene in the DF-PBFB and controls. The evolution of the number of copies of the 16S rRNA gene (in the logarithmic scale) in the DF-PBFB and controls during faecal fermentations, relative to the target bacterial groups, is presented in Figure 2 and Table 3.

There are a lack of data about the impact of the consumption of fermented cereals on the human gut microbiota [65,66], with the study of native cereals being more common in general, or of some compounds that are generated or augmented during fermentation, such as EPS or polyphenols [66]. However, there are some research studies that are worthy to be mentioned. One of them, carried out by Sori et al. (2023), studied the effects of two types of millets (Kodo and Kutki millets) on the human gut microbiota in terms of its potential modulation and metabolites produced [67]. Both millets increased the levels of Firmicutes and Actinobacteria and reduced the levels of Bacteroidetes. In addition, the correlation analysis between the gut microbiota and the metabolome demonstrated a link between beneficial metabolites (e.g., acetate, butyrate, propionate, and formate, among others) and the presence of *Caproiciproducens*, *Lactobacillus*, *Veillonella*, *Blautia*, *Faecalibacterium*, and *Prevotella* [67]. Additionally, Liu et al. (2021) [68], in a *post hoc* analysis of a 12-week randomised controlled trial in Chinese adults with *Helicobacter pylori* infection, studied the impact of a high intake of whole grain rye products containing fermented rye bran on the gut microbiota and SCFA composition compared to an intake of refined wheat-based products. The authors found increased levels of *Romboutsia* (a member of the genus *Clostridium*) and a lower abundance of *Bilophila* (bile-resistant opportunistic pathogen) in the fermented rye bran group compared to the refined wheat group. The potential gut microbiota modulation, with increased levels of *Bifidobacterium*, *Lactobacillus*, and *Bacteroides* and a reduction in *Clostridium*, due to the consumption of whole grain oat products has been being demonstrated by in vitro fermentation models and animal studies [69]. The prebiotic effects of oat bran were also observed, but not for its bioactive compounds, β-glucans or polyphenols, separately, suggesting that this effect might come from the harmonious combination of all oat compounds and cereal matrix; such synergy between compounds has also been explored for other food matrices [69]. Also, Yan et al. (2022) observed that administering fermented rice buckwheat (by *Bacillus sp.* DU-106 and *L. plantarum)* positively modulated the gut microbiota in mice. This was achieved by reducing the Firmicutes-to-Bacteroidetes ratio, increasing the abundance of SCFA-producing bacteria such as *Bacteroides*, *Lactobacillus*, and *Blautia*, and enhancing overall SCFA levels [70]. In 2023, an integrative review carried out by Fabiano et al. showed that the literature is conclusive about the prebiotic effects of oats, showing that oat intake resulted in increased levels not only of *Lactobacillus*, *Bifidobacterium* and *Akkermansia*, but also of butyrate, propionate, acetate, and valeric acid [71].

On the other hand, the review by Dimidi et al. (2019) [65] summarises the main publications on this topic focused on fermented cereals, such as kefir, kombucha, tempeh, natto, kimchi and sourdough bread. The authors concluded that kefir is the most commonly investigated fermented food concerning gut microbiota impact, and satisfactory results have been demonstrated to promote the growth of healthy bacterial communities, through *H*. *pylori* eradication as well as posing benefits for lactose malabsorption patients [65]. Natto and kimchi have also revealed increased levels of *Bifidobacterium* or *Lactobacillus* in in vivo studies, but no evidence exists for kombucha, and some controversial results were found for sourdough bread. A recent study explored the impact of the consumption of fermented foods, with a focus on plants with modifications in the human gut microbiome, using a subset of the American Gut Project cohort as the sample, finding an overall difference between the gut microbial communities of consumers and non-consumers, and showed that consumers had higher concentrations of the bacterial groups that are more associated with fermented foods [72].

- *Bifidobacterium* spp.

*Bifidobacterium* spp. belongs to the Actinobacteria phylum, one of the five most abundant phyla in the human gut microbiota. Concerning the present results, it is possible to observe a positive impact from the DF-PBFB on the number of copies of the 16S rRNA gene after 6 h of fermentation (*p* ≤ 0.05). However, this effect is not maintained until the end of the process, due to the high variability resulting from one of the five donors, whose faeces showed a different behaviour during the 24 h. If this donor was be excluded, a consistent effect would have been observed over time. Nevertheless, in terms of statistics, that donor was not considered an outlier, which is the reason that it was included in the final sample. Therefore, it seems that DF-PBFB has a potential bifidogenic effect. This trend was partially followed by FOS, which had also shown a significant increase during the first 6 h, and the positive impact was maintained over the fermentation time. Nevertheless, the increment during the first 6 h was more impactful for the DF-PBFB, but no statistical differences were observed. For the negative control, no differences were observed over time. When comparing the increment in the number of copies of the 16S rRNA gene between the DF-PBFB and the controls, DF-PBFB and FOS had similar increments over time (with the exception at 24 h), which were higher than the negative control; however, these were not statistically different. FOS is a recognisable prebiotic [26] that stimulates the growth of *Bifidobacterium*, and this fructooligosaccharide has been used as a positive control in the same type of in vitro studies, also showing a positive impact on the target bacterial group [45,73,74]. The presence of EPS in the DF-PBFB might be behind this bifidogenic effect, as microbial EPS have been hypothesised as having important prebiotic effects depending on their molecular mass and type of bonds [75,76,77]. It is expected that EPS predominantly comprising α-1,6-glycosidic bonds will escape digestion by the intestinal lumen enzymes, thereby reaching the colon partly intact to be metabolised by the microbiota present [77].

- *Lactobacillus* spp.

As previously mentioned, for the purpose of this manuscript, *Lactobacillus* refers to the species previously classified as *Lactobacillus*, prior to the reformulation of the genus in March 2020. The baseline presence of *Lactobacillus* spp. in the sampled faeces was lower compared to other bacterial groups. *Lactobacillus* belong to the phyla Firmicutes, one of the major resident phyla in the gut microbiota; however, the genus represents only 0.3% of the total bacteria in the colon, and 6% in the duodenum [78]. Nevertheless, *Lactobacillus*, even in smaller concentrations, are correlated with the absence of several diseases [78]. When in the presence of the DF-PBFB, despite the fact that no increase in the genus was observed over time, significantly higher concentrations compared to the positive and negative controls were noticed from the beginning and over 12 h of fermentation (*p* ≤ 0.05). This shows the potentiality of the DF-PBFB as a carrier of *Lactobacillus* into gut colonisation. Consequently, the increased number of lactobacilli in the gut microbiota may result in potential health benefits, since they are health-promoting bacteria. Regarding the positive control, an increase in those numbers were observed; however, there were no statistically significant differences in fermentation, given the high variability between individuals. On the contrary, in the negative control, no changes were observed over time. This variability could be potentially reduced by recruiting a wider volunteer sample; nevertheless, this number of individuals (between 2 and 6) is commonly used in these type of studies [45,73,79,80]. In the study of Amaretti et al. 2020 [76], in which the prebiotic effect of low-molecular mass EPS was evaluated and compared with inulin, the genus *Lactobacillus* did not reach the limit of detection, either for the EPS or inulin, suggesting that both substrates do not support their growth. Also, Connolly et al. (2012) [81], using an in vitro model, tested raw/toasted/partially toasted wheat flakes, FOS, and cellulose, and only raw wheat promoted the growth of this genus.

- *Clostridium leptum* subgroup and *Faecalibacterium prausnitzii*

*Clostridium leptum* subgroup, also known as *Clostridium* cluster IV, represents between 16 and 25% of the faecal microbiota in adult humans, and it includes *Faecalibacterium prausnitzii* and certain species of *Eubacterium* and *Ruminococcus* [82]. In this study, the DF-PBFB and the positive control have induced a decrease in the numbers of the *C*. *leptum* subgroup and *F*. *prausnitzii*; however, these differences were not considered statistically significant. When no carbon source was added (negative control), the results followed the same trend. In the study of Mao et al. (2015) [83] that evaluated the effects of FOS on gut bacteria in mice, FOS in low doses (5%), similarly to the present study (2%), only resulted in higher concentrations of *Clostridium* after 4 wk of feeding. Nevertheless, those increases were not significant, and their abundance remained low. Similarly to the present results, in the work of Amaretti et al. (2020) [76], *F*. *prausnitzii* was not positively affected by inulin over 24 h fermentation. On the contrary, the work of Ramirez-Faria et al. (2009) [84] revealed the increase in F. p*rausnitzii* in adult faecal samples after the ingestion of inulin (10 g/d) for a 15 d period. These bacteria have been associated with inflammatory conditions, namely inflammatory bowel diseases, with Crohn’s disease and ulcerative colitis patients demonstrating *C. leptum* group species numbers reduced compared to healthy controls [82]. From this perspective, it would have been desired that the DF-PBFB had a positive impact on *Clostridium leptum* subgroup numbers.

- *Roseburia* spp.

For this bacterial group, belonging to the Clostridium cluster XIVa, a decrease over time was observed in the presence of the DF-PBFB (*p* ≤ 0.05). This was similar to the positive control (FOS), although for the latter, no statistically significant differences were observed until 12 h of fermentation. For the negative control, despite the observed reduced levels, no differences were observed in statistical terms (*p* > 0.05). On the contrary, the study of Vanegas et al. (2017) [85] observed that the consumption of whole grain wheat had a positive impact compared to the control, i.e., refined wheat, on *Roseburia* abundance after a follow-up of 8 wk. Also, in the study of Mota de Carvalho et al. (2019), in which the in vitro assessment of the impact of an edible insect on the gut microbiota was carried out, *Roseburia*’s number of copies of the 16S rRNA gene showed an increase while fermenting FOS over 24 h, and experienced a decrease after that period and a decrease during the fermentation of the digested *Tenebrio molitor* insect flour [45]. *Roseburia* is recognised for using resistant starch as an energy source [86], mostly of type 3; it was also found to utilise mucin [87]. This might suggest that *Roseburia* bacteria prefer to metabolise higher molecular mass compounds.

- *Bacteroides* spp.

*Bacteroides* are saccharolytic fermenters known for utilising complex carbohydrates [65], in which genus of healthy and harmful bacteria are found [26]. In the present study, a decrease in the number of copies of the 16S rRNA gene over time was observed in the presence of the DF-PBFB; however, it was not considered statistically different (*p* > 0.05). Nevertheless, in the presence of the DF-PBFB, absolute values were significantly lower when compared to both controls, suggesting that a bacterial competition may be present between the endogenous LAB strains and the *Bacteroides* genus bacteria. Connolly and colleagues (2012) [81] observed an increase in *Bacteroides* spp., which was promoted by the toasted wheat. Despite the higher values obtained when fermenting FOS, no significant differences were considered. On the contrary, in other studies, fermenting FOS resulted in *Bacteroides* having a lower number of 16S gene copies [45,73,74]. Some studies revealed that, when in the presence of inulin, no differences are observed over time [76,84], while in the presence of the dextran that is synthetized by *W*. *confusa*, *Bacteroides* spp. increased its numbers, a trend that was not observed in the present study. Also, Zhu and collaborators (2021) observed that *Lactiplantibacillus*-fermented black rye showed to have a modulatory effect on high-fat diet-induced intestinal microbiota dysbiosis by increasing the relative abundance of Bacteroides [88].

The previous bacterial enumeration results were supported by the acidification observed in the DF-PBFB’s and FOS’s fermentation, along with the sugars consumption and the subsequent production of organic acids (Table 4). The major organic acid produced was lactic acid, followed by the SCFA acetic, propionic, and butyric acids. Succinic acid was also considerably produced. Production profiles were similar for both the positive control (FOS) and DF-PBFB by 12 h and 24 h fermentation (*p* > 0.05).

Lactic acid, which is mainly produced by LAB despite not being considered a SCFA, has several benefits for the host. Its major production over time (*p* ≤ 0.05) is justified by the higher levels observed for the *Lactobacillus* genus. For the DF-PBFB, a progressive increase (*p* ≤ 0.05) until 12 h of fermentation was observed, while for FOS, the more impactful period was the first 6 h (*p* ≤ 0.05), remaining constant until the end of fermentation (*p* > 0.05). Lactate has been associated with several bioactive activities, besides its well-recognised organoleptic and antimicrobial properties, among which the capacity to reduce innate response activation and proinflammatory cytokine levels can be highlighted, along with the ability of reinforce gut barrier function [89].

In both substrates, FOS and the DF-PBFB, the production of acetate was observed over time. However, during the first 6 h, the fermentation of FOS resulted in almost twice as high levels of production as those observed for the DF-PBFB; nevertheless, acetate concentrations after 24 h were considered similar between both. The bacterial groups mostly responsible for the production of acetic acid are Bacteroidetes, *Bifidobacterium*, and also Firmicutes [87]. Acetate can be produced either via acetil Co-A or H_2_, CO_2_, or formate, using hexoses or pentoses as source of energy [87]. This SCFA has been associated with weight management; however, controversial results, depending on the species or the GI location where the acetate is released, have been achieved [90]. Other effects that derive from acetate production act on appetite regulation, adipose tissue lipolysis regulation, and also on blood pressure regulation [91]. It has also been demonstrated that acetate promotes plasma membrane re-localization in MCT-1 (a SCFA transporter), triggers changes in glucose metabolism, and induces apoptotic cell death in colorectal cancer cells, involving lysosomal membrane permeabilization and the release of Cathepsin D, which is associated with mitochondria dysfunction [92].

Once acetate is produced, this SCFA is also available for cross-feeding interactions, resulting in the production of butyrate, via butyryl–CoA:acetate CoA-transferase, by butyrate-producing colonic bacteria, such as the bacteria belonging to the *Clostridium leptum* subgroup, with *Faecalibacterium* being one of the most relevant [82,87,93]. Nevertheless, butyrate is also generated through the formation of butyryl-P from carbohydrates [87]. Butyrate was the SCFA found in the lowest concentrations in the present study. Such a trend was expected, since the growth of butyrate-producing bacterial groups was promoted neither by FOS, nor the DF-PBFB. Another reason that might justify the lower production of butyrate is the low pH reached after 6 h of fermentation, since butyrogenic bacteria are sensitive at this pH [87,93]. In the negative control, in which a not-so-low pH was obtained, butyrate was produced in higher concentrations. Butyrate has been revealed to have a significant role on the immune system, beneficial effects on the GI tract, namely the epithelial barrier, and a colorectal cancer protective role. It has also been associated with an increase in satiety [91,93].

Propionate is another SCFA with interesting health promotion properties. In the present study, the concentrations of propionate increased in the presence of both the DF-PBFB and the positive control. The main bacterial groups responsible for its production are Bacteroidetes, but also Firmicutes and *Clostridium* cluster IV, and different routes are used for propionate formation. Propionate is mainly generated via the succinate pathway; however, the acrylate pathway, in which lactates are involved, and the propanediol pathway, in which deoxyhexose sugars are utilised, are alternatives [87]. The positive health effects associated with this SCFA are linked to the reduction in food intake and the prevention of long-term weight gain, which is on the one hand due to the modulation of gut hormones release, and on the other hand, direct neural gut–brain signalling via free-fatty acids receptors [91]. Moreover, it has been associated with an improvement in pancreatic function and a reduction in hepatic lipid storage [91,94].

Succinate is not a SCFA, but it can be produced during microbial fermentation in the gut. Succinic acid is usually generated by the reversal of partial tricarboxylic acid cycle reactions, and this cycle is the main pathway to produce the metabolic intermediate succinate [76]. Succinate is traditionally associated with a less positive effect on the human host, specifically acting as a proinflammatory on the regulation of local stress, tissue damage, and immune response [95]. However, this organic acid has demonstrated an interesting performance in the regulation of intestinal gluconeogenesis and thermogenesis, and also plays a key role in obesity-associated inflammation [94,95]. Considering the present results, its concentrations increased after 6 h of fermentation in the presence of FOS but not with DF-PBFB, given the initial quantities present. Moreover, in the study of Amaretti et al., succinic acid was produced over the first 6 h, and decreased over the remaining period until 24 h of fermentation [76].

In the negative control, an increase in acetate and butyrate (which was only significantly different at 24 h), and propionate (which was only significantly different after 12 h) production was observed. The propionate increase might be due to the high presence of *Bacteroides*, which contribute to most of propionic acid produced in the colon [96]. The observed increase in butyrate and acetate levels was not expected given the lack of growth in the main producing strains of the bacterial groups, *Clostridium leptum* subgroup and *Bifidobacterium*, respectively.

Gut microbiota modulation is transient, given that it is primarily influenced by dietary intake and the availability of non-digestible carbohydrates in the microbiota, which affect the balance between saccharolytic fermentation and proteolytic fermentation [91]. Consequently, people with different dietary patterns, which are intricately linked to living conditions, place of residence, financial availability, and purchasing capacity, are expected to have different gut microbiota composition profiles. The study of De Fillipo and colleagues (2010) compared the faecal microbiota of European children, with a westernised diet, to that of rural children from Burkina Faso, with a diet rich in fibre and low in fat and protein, observing that African children had an enrichment in Bacteroidetes, *Prevotella*, Xylanibacter, and general SCFA, while the European children showed higher representativeness of Firmicutes and Enterobacteriaceae (Shigella and Escherichia) [97]. Besides individuals’ diets, the gut microbiota is influenced by other intra-individual factors, such as infant transitions (birth- and diet-related), age, the presence of diseases, and the consumption of antibiotics [98]. Inter-individual factors, such as ethnicity, gender, body mass index, dietary habits, and cultural habits, are also linked to gut microbiota differences [98]. In the present study, factors such as age, ethnicity, the presence of diseases, and the consumption of antibiotics did not have any influence, given the transversality between donors. However, while gender, body mass index, and dietary habits differed within the sample, no specific influences should be inferred given the small sample size, which is one of the limitations of the present research work. The lack of stirring during fermentation, and also of pH control, did not allow a more realistic recreation of the intestinal conditions. Nonetheless, in vitro studies like the one reported herein are important and useful for the early development of potentially prebiotic food products, contributing towards an ultimate validation of prebiotic activity, which can only be concluded from in vivo feeding studies.

## 4. Conclusions

The present research work describes the development of a novel functional whole grain-fermented beverage, revealing increased levels of phenolic compounds and antidiabetic potential. The promotion of *Bifidobacterium* growth and the increase in the numbers of lactobacilli in the in vitro assay demonstrates the potential positive effects on the probiotic flora on the developed fermented plant-based beverage. Therefore, there might be a beneficial impact resulting from the consumption of this innovative fermented product on blood glucose levels, and a positive modulation of the human gut microbiota. While these findings are promising, it is important to highlight their preliminary nature. Given the variability observed among participants, further research is needed to confirm these effects. Larger, long-term cohort studies with more robust experimental designs and diverse populations are essential to establish the consistency and sustainability of the observed changes in microbiota composition. Only through such studies can the full potential of this fermented beverage be realised and its health benefits definitively determined.

The envisaged technology, fermentation, is already practised in developing countries; however, this practice is not under controlled conditions, posing a health hazard to the consumers. In this sense, the use of the present technology brings quality, safety, and shelf-life advantages to a very nutritious cereal matrix, ginger millet, which is shaping up to be a potential solution to malnutrition. Additionally, this study highlights the novel use of a unique combination of probiotic and techno-functional cultures to ferment finger millet, which is an underutilised substrate despite its rich nutrient profile and favourable environmental characteristics. While existing literature has explored the modulation of the gut microbiota by native cereals, research specifically examining fermented cereals—particularly finger millet—remains limited. By addressing this gap, our findings offer valuable insights into the potential of LAB-fermented finger millet as a functional food for enhancing gut health.

## Figures and Tables

**Table 1:** Primers sequences targeting bacterial groups and respective optimal annealing temperature and genomic DNA standards, and respective genome size (base pairs) and the number of copies of the 16S RNA gene.

Target Group	Primers Sequence (5′-3′) (F: Forward; R: Reverse)	Optimal Annealing Temperature (°C)	Microorganism	Strain Reference	Genome Size (Base Pairs)	Copies of 16S RNA Gene
*Bifidobacterium* spp.	F: CGC GTC YGG TGT GAA AGR: CCC CAC ATC CAG CAT CCA	62	*Bifidobacterium longum* subsp. *infantis*	DSM 20088 (S12)	2,832,748	4
*Lactobacillus* spp. ^1^	F: CAC CGC TAC ACA TGG AG R: AGC AGT AGG GAA TCT TCC A	59	*Lacticaseibacillus rhamnosus*	Lcr35	2,937,400	5
*Clostridium leptum* subgroup	F: GCA CAA GCA GTG GAG TR: CTT CCT CCG TTT TGT CAA	57.5	*Clostridium leptum*	DSM 753 (VPI T7-24-1)	3,270,109	2
*Roseburia* spp.	F: TAC TGC ATT GGA AAC TGT CG R: CGG CAC CGA AGA GCA AT	60	*Roseburia hominis*	DSM 16839 (A2-183)	3,592,125	4
*Faecalibacterium prausnitzii*	F: GGA GGA AGA AGG TCT TCG G R: AAT TCC GCC TAC CTC TGC ACT	60	*Faecalibacterium prausnitzii*	DSM 17677 (A2-165)	3,214,418 ^2^	6 ^2^
*Bacteroides* spp.	F: ATA GCC TTT CGA AAG RAA GAT R: CCA GTA TCA ACT GCA ATT TTA	54.5	*Phocaeicola vulgatus* (former *Bacteroides vulgatus*)	DSM 1447	5,163,189	7

^1^ ‘*Lactobacillus*’ refers to those species previously classified as *Lactobacillus* prior to the reformulation of the genus in March 2020. ^2^ These parameters refer to *Faecalibacterium prausnitzii* SL3/3, given the lack of information on the strain used.

**Table 2:** The total phenolics content (TPC) (mg GAE^1^/kg of plant-based functional beverage (PBFB)), antioxidant activities (DPPH, mg TE^2^/kg of PBFB; ABTS mg AAE^3^/kg of PBFB), and α-glucosidase inhibitory activity of dry-milled finger millet unfermented slurry and the fermented PBFB, carried out by *L*. *plantarum* 299v co-cultured with *W*. *confusa* 2LABPT05, at 30 °C and 200 rpm, over 8 h, in an orbital incubator.

	Unfermented Slurry	Fermented PBFB (F-PBFB)
TPC (mg GAE ^1^/kg PBFB)	181 ± 11 ^a^	244 ± 11 ^b^
DPPH (mg TE ^2^/kg PBFB)	180 ± 11 ^a^	153 ± 11 ^b^
ABTS (mg AAE ^3^/kg PBFB)	239 ± 15 ^a^	238 ± 8 ^a^
α-glucosidase inhibitory activity (%)	14 ± 2 ^a^	21 ± 2 ^b^

^1^ GAE: gallic acid equivalent; ^2^ TE: Trolox equivalent; ^3^ AAE: ascorbic acid equivalent. For a given parameter, different letters indicate significant differences (*p* ≤ 0.05), between samples, using the paired T-test or Wilcoxon test.

**Table 3:** Quantification of the bacterial groups (expressed as log (16S rRNA gene copies/ng of DNA); mean ± standard deviation) detected by qPCR in faecal samples (negative control (C−), positive control (C+; FOS), digested fermented PBFB (DF-PBFB)) of five donors.

		Log (16S rRNA Gene Copies/ng DNA)														
		C−					C+ (FOS)					DF-PBFB				
		Absolute Concentration				Increment (+)/Reduction (−)	Absolute Concentration				Increment (+)/Reduction (−)	Absolute Concentration				Increment (+)/Reduction (−)
*Bifidobacterium* spp.	0 h	4.3	±	0.7	^a,x^	n.a. ^1^	3.9	±	0.7	^a,x,y^	n.a.	3.1	±	0.9	^a,y^	n.a.
	6 h	4.3	±	0.3	^a,x^	+1.4	5.2	±	0.5	^b,x^	+33.1	4.2	±	0.3	^b,x^	+41.3
	12 h	4.1	±	0.3	^a,x^	−1.6	5.1	±	0.4	^b,x^	+31.7	4.3	±	0.2	^b,x^	+37.5
	24 h	4.5	±	0.2	^a,x,y^	+6.0	5.2	±	0.5	^b,x^	+32.8	4.1	±	0.2	^a,b,y^	+31.7
*Lactobacillus* spp.	0 h	2.2	±	0.6	^a,x^	n.a.	1.9	±	0.9	^a,x^	n.a.	5.3	±	0.2	^a,y^	n.a.
	6 h	2.2	±	0.8	^a,x^	−1.9	2.6	±	1.6	^a,x^	+38.2	5.1	±	0.2	^a,y^	−3.9
	12 h	2.0	±	1.0	^a,x^	−10.7	2.6	±	1.6	^a,x^	−13.9	5.1	±	0.3	^a,y^	−4.5
	24 h	2.1	±	0.9	^a,x^	−7.4	3.0	±	1.6	^a,x,y^	−18.0	5.1	±	0.2	^a,y^	−4.3
*Clostridium* *leptum* subgroup	0 h	4.1	±	0.4	^a,x^	n.a.	4.0	±	0.5	^a,x^	n.a.	2.8	±	0.7	^b,y^	n.a.
	6 h	3.6	±	0.3	^a,x^	−12.5	3.5	±	0.1	^a,x^	−12.5	2.4	±	0.2	^b,y^	−15.4
	12 h	3.5	±	0.2	^a,x^	−14.5	3.5	±	0.2	^a,x^	−13.9	2.1	±	0.4	^b,y^	−24.0
	24 h	3.4	±	0.4	^a,x^	−16.4	3.3	±	0.2	^a,x^	−18.0	2.1	±	0.5	^b,y^	−23.0
*Roseburia* spp.	0 h	3.4	±	0.7	^a,x^	n.a.	3.5	±		^a,x^	n.a.	2.3	±	0.6	^a,y^	n.a.
	6 h	2.9	±	0.4	^a,x^	−11.1	2.6	±	0.2	^a,b,x,y^	−27.9	1.7	±	0.4	^a,b,y^	−27.7
	12 h	2.5	±	0.4	^a,x^	−22.8	2.6	±	0.2	^a,b,x^	−26.8	0.7	±	0.5	^b,y^	−66.3
	24 h	2.4	±	0.6	^a,x^	−24.8	2.4	±	0.2	^b,x^	−31.2	<LOQ ^2^				n.a.
*Feecalibacterium praunitzii*	0 h	4.1	±	1.0	^a,x^	n.a.	4.2	±	1.1	^a,x^	n.a.	2.8	±	1.0	^a,x^	n.a.
	6 h	3.8	±	0.6	^a,x^	−9.1	3.6	±	0.9	^a,x^	−15.5.	2.7	±	0.7	^a,x^	−2.3
	12 h	3.5	±	0.5	^a,x^	−15.5	3.6	±	0.9	^a,x^	−14.6	2.6	±	0.7	^a,x^	−6.4
	24 h	3.2	±	0.5	^a,x^	−23.7	3.4	±	0.7	^a,x^	−18.6	2.2	±	0.9	^a,x^	−18.7
*Bacteroides* spp.	0 h	3.9	±	0.7	^a,x^	n.a.	4.1	±	0.6	^a,x^	n.a.	1.3	±	0.6	^b,y^	n.a.
	6 h	3.9	±	0.8	^a,x^	−0.5	3.4	±	0.6	^a,x^	−16.3	1.3	±	0.3	^b,y^	2.0
	12 h	4.3	±	0.6	^a,x^	+6.8	3.9	±	0.8	^a,x^	−2.4	0.5	±	0.3	^b,y^	−45.7
	24 h	4.7	±	0.6	^a,x^	+15.9	3.7	±	0.8	^a,x^	−5.2	<LOQ				n.a.

For each bacterial group, for a given sample, different letters (a, b, and c) indicate significant differences between times (*p* ≤ 0.05). For a given time, different letters (x, y, and z) indicate significant differences between samples (*p* ≤ 0.05), using one-way ANOVA. The increment/reduction values are expressed in %. ^1^ n.a.: not applicable. ^2^ LOQ: limit of quantification.

**Table 4:** The concentrations (mean in g/kg *±* standard deviation) of consumed sugars and produced organic acids during fermentation over 24 h in faecal samples (negative control (C−), positive control (C+; FOS), digested fermented PBFB (DF-PBFB)) of five donors. The presented values are the mean of the five donors, considering one injection for each sample.

	Time (h)	C−		C+ (FOS)		DF-PBFB	
**pH**	0	6.947 ± 0.008	^a,x^	6.94 ± 0.02	^ax^	6.61 ± 0.02	^ax^
	6	6.6 ± 0.1	^a,x^	4.5 ± 0.3	^b,y^	4.8 ± 0.4	^b,y^
	12	6.58 ± 0.07	^a,x^	4.2 ± 0.3	^b,y^	4.6 ± 0.3	^b,y^
	24	6.6 ± 0.1	^a,x^	4.0 ± 0.3	^b,y^	4.5 ± 0.3	^b,z^
		**Sugars and organic acids (g/kg)**					
**Sucrose**	0	<LOD		<LOD		3.1 ± 0.1	^a^
	6	<LOD		<LOD		2.1 ± 0.3	^b^
	12	<LOD		<LOD		2.1 ± 0.5	^b^
	24	<LOD		<LOD		1.9 ± 0.3	^b^
**Glucose**	0	<LOD		<LOD		2.9 ± 0.1	^a^
	6	<LOD		<LOD		2.3 ± 0.3	^b^
	12	<LOD		<LOD		2.1 ± 0.3	^b^
	24	<LOD		<LOD		2.0 ± 1.4	^b^
**Fructose**	0	<LOD		2.1 ± 0.3	^a,x^	1.8 ± 0.2	^a,x^
	6	<LOD		2.4 ± 1.0	^a,x^	1.3 ± 0.5	^a,x^
	12	<LOD		2.4 ± 0.9	^a,x^	1.0 ± 0.4	^a,y^
	24	<LOD		2.6 ± 1.2	^a,x^	1.0 ± 0.3	^a,y^
**Succinic acid**	0	0.20 ± 0.04	^a,x^	<LOD		0.7 ± 0.1	^a,y^
	6	0.17 ± 0.03	^a,x^	0.6 ± 0.3	^a,y^	0.8 ± 0.1	^a,y^
	12	0.13 ± 0.04	^a,x^	0.6 ± 0.4	^a,y^	0.8 ± 0.1	^a,y^
	24	<LOD		0.6 ± 0.2	^a,x^	0.8 ± 0.2	^a,x^
**Lactic acid**	0	<LOD		<LOD		0.9 ± 0.1	^a^
	6	<LOD		1.8 ± 0.6	^a,x^	1.6 ± 0.3	^a,x^
	12	<LOD		2.0 ± 0.9	^a,x^	2.3 ± 0.8	^b,x^
	24	<LOD		2.2 ± 0.9	^a,x^	2.3 ± 0.7	^b,x^
**Acetic acid**	0	0.0485 ± 0.007	^a^	<LOD		<LOD	
	6	0.17 ± 0.06	^a,b,x^	1.0 ± 0.5	^b,y^	0.59 ± 0.07	^b,x,y^
	12	0.40 ± 0.07	^a,b,x^	0.9 ± 0.3	^b,x^	0.8 ± 0.2	^b,x^
	24	0.59 ± 0.06	^b,x^	1.0 ± 0.3	^b,x^	0..8 ± 0.2	^b,x^
**Propionic acid**	0	0.04 ± 0.03	^a^	<LOD		<LOD	
	6	0.16 ± 0.03	^a,x^	0.3 ± 0.1	^a,y^	0.4 ± 0.2	^a,z^
	12	0.25 ± 0.05	^b,x^	0.27 ± 0.09	^a,x^	0.4 ± 0.1	^a,x^
	24	0.37 ± 0.05	^b,x^	0.26 ± 0.07	^a,x^	0.4 ± 0.1	^a,x^
**Butyric acid**	0	<LOD		<LOD		<LOD	
	6	0.16 ± 0.07	^a,x^	0.3 ± 0.1	^a,y^	0.19 ± 0.07	^a,y^
	12	0.29 ± 0.09	^a,b,x^	0.2 ± 0.1	^a,x^	0.20± 0.05	^a,x^
	24	0.42 ± 0.09	^b,x^	0.2 ± 0.1	^a,y^	0.20 ± 0.06	^a,y^

For each bacterial group, for a given sample, different letters (a, b, and c) indicate significant differences between times (*p* ≤ 0.05), and for a given time, different letters (x, y, and z) indicate significant differences between samples (*p* ≤ 0.05), using one-way ANOVA. LOD: limit of detection (sucrose: 0.05 g/L; glucose: 0.1 g/L; fructose: 0.1 g/L; succinic acid: 0.05 g/L; lactic acid: 0.05 g/L; acetic acid: 0.05 g/L; propionic acid: 0.01 g/L; butyric acid: 0.01 g/L).

## Data Availability

The original contributions presented in the study are included in the article, further inquiries can be directed to the corresponding author.
